# Advances in organoid technology for veterinary disease modeling

**DOI:** 10.3389/fvets.2023.1234628

**Published:** 2023-10-18

**Authors:** Bo Chen, Ronald Francis Slocombe, Smitha Rose Georgy

**Affiliations:** Section of Anatomic Pathology, Melbourne Veterinary School, Faculty of Science, University of Melbourne, Werribee, VIC, Australia

**Keywords:** organoids, disease modeling, *in vitro* model, veterinary disease, infectious disease, cancer, metabolic disease, inflammatory disease

## Abstract

Organoids are *in vitro* organ-like structures that faithfully recapitulate many characteristics of a specific organ. During the past decades, major progress has been accomplished in establishing three-dimensional (3D) culture systems toward stem cell-derived organoids. As a significant technological breakthrough, these amazing 3D organoid constructs bridge the conventional 2D *in vitro* models and *in vivo* animal models and provide an unprecedented opportunity to investigate the complexities of veterinary diseases ranging from their pathogenesis to the prevention, therapy, or even future organ replacement strategies. In this review, we briefly discuss several definitions used in organoid research and highlight the currently known achievements in modeling veterinary diseases, including infectious and inflammatory diseases, cancers, and metabolic diseases. The applications of organoid technology in veterinary disease modeling are still in their infancy stage but the future is promising.

## 1. Introduction

The transition of *in vitro* models from the traditional monolayer cell culture to a three-dimensional (3D) system certainly represents the most critical innovation of the last decade for *in vitro* studies. One of the most significant advancements in the 3D models is the generation of organoids or ‘mini organs on a dish’ as a frontier technology (1). In recent years, there is an increasing number of research publications where the organoid model is applied to study diseases of veterinary importance, but in comparison to medical research, its potential is not yet fully explored for veterinary medicine.

The first usage of the term “organoid,” which means “resembling an organ,” was in 1946 when the authors described a cystic teratoma (2). Teratomas develop from pluripotent stem cells (PSCs) of the germ line and organize a variety of specific organotypic structures such as skin, nerve, intestine, bone, and tooth, owing to the processes of recapitulation of cell segregation and fate specification. Now the term “organoid” is generally accepted to mean a 3D aggregation of organ-specific cell types. Like teratomas, organoids develop from stem cells or organ progenitors (e.g., intestinal crypts) and self-organize through two processes of recapitulation of cell segregation and fate specification like *in vivo* development and growth (1). Self-organization is essential in organoids as a distinction from two-dimensional cultures.

**Figure 1:**
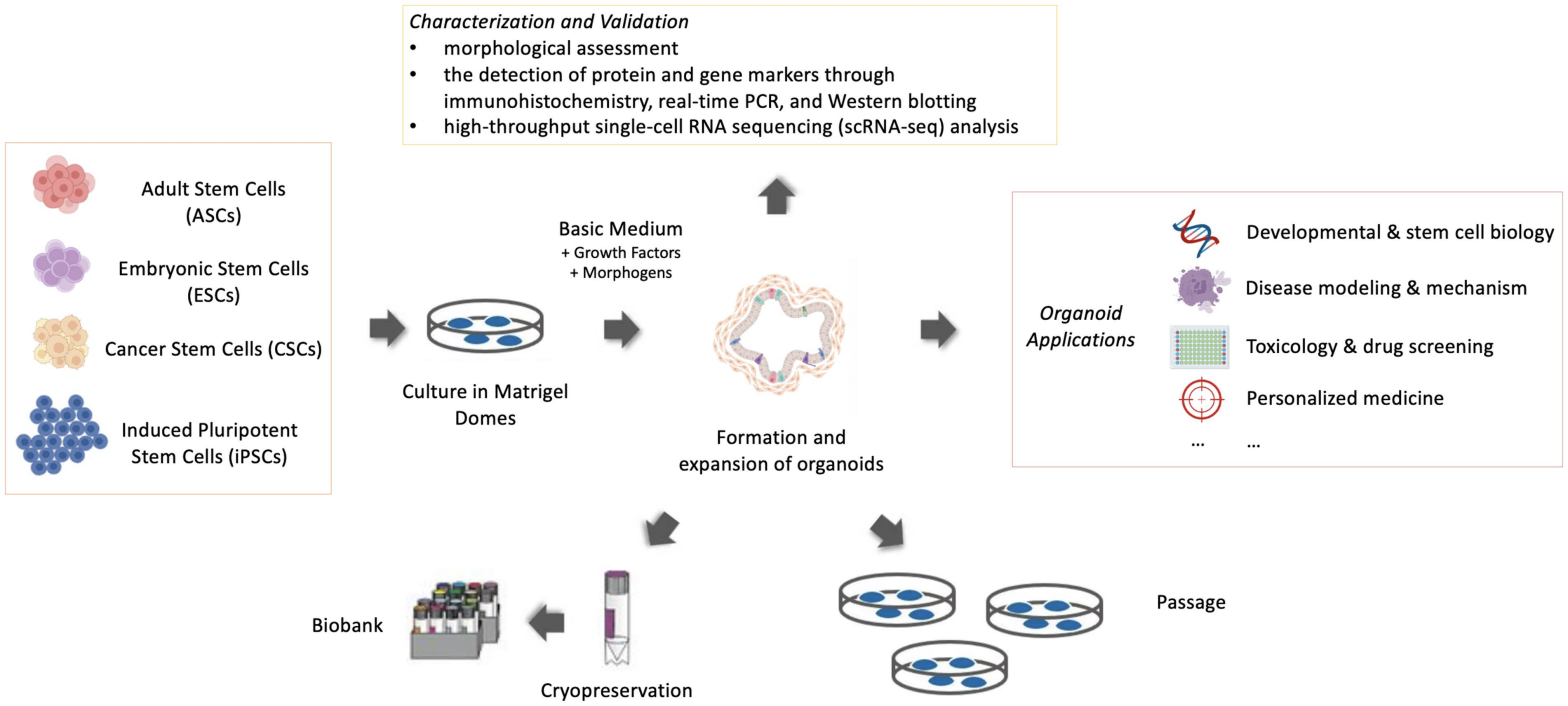
Schematic representation of organoid culture, maintenance and application (Created with BioRender.com).

The significant steps to generate an organoid are the initial proliferation of stem and precursor cells in a proper environment. Derivation from stem cells is a critical feature in the definition of an organoid and differentiates organoids from tissue explants, which are derived from an organotypic culture of cells or small segments of tissue (3). The stem cells included embryonic stem cells (ESCs), induced pluripotent stem cells (iPSCs) or organ-specific adult stem cells (ASCs) (4). ESCs are derived from early-stage embryos. Induced pluripotent stem cells (iPSCs) are a type of stem cells that are genetically reprogrammed from adult somatic cells and exhibit many similarities to ESCs in many properties (4). ASCs in principle are obtained from ‘mature’ or adult tissue, but they are not necessarily from adult animals but can be from juveniles or even from advanced embryos (1, 5). Cancer stem cells (CSCs) can also be considered one kind of ASCs and are able to produce 3D tumoroids in appropriate environments (6). ASC-derived organoids are intrinsically programmed with their location-specific functions (7), making them more “adult-like” than organoids derived from ESC, which retain tissue-associated mesenchymal cells (Figure 1).

Three-dimensional (3D) cell culture methods represent any culture of cells that recapitulates their 3D organization and/or cell–cell and cell-matrix interactions *in vitro* (8). 3D cell cultures of more cell types are often referred to as co-culture (9). When stem cells are supplied with proper culture conditions and scaffolding to promote their growth and survival, they can generate miniature organ models known as organoids or spheroids that mimics cell types, structure, and functions (10, 11). The use of the terms ‘organoid’ and ‘spheroid’ can vary or overlap in different published works. Some works, especially in the early stage of organoid research, prefer to use the term ‘organoids’ to indicate both, or consider ‘spheroid’ as one simple, primitive, or intermediate type of ‘organoid’ (11). Other opinions have a more rigid definition that spheroids are often referred to as scaffold-free, simple-cell aggregates with a single-cell origin (12, 13). Herein, a more explicit definition of ‘organoid’ was proposed based on three essential requirements that need to be fulfilled: first, an organoid must contain more than one cell type of the organ it models; second, it must recapitulate some of the specific functions of that organ; and third, the cells should have a similar spatial organization as the organ (1). Since organoids represent a higher order of complexity and recapitulate their parent organ more closely than spheroids, researchers are more inclined toward organoid technology for disease modeling and optimizing drug discovery and personalized medicine (14). Alternatively in cancer research, spheroids that can be generated from cancer cell lines or tumor fragments are preferred, because they still closely mimic the main features of solid tumors’ structures and functions as simple clusters of freely floating cancer stem cell aggregates (15).

Ambiguities also have arisen in gastrointestinal organoid nomenclature, when mentioning “enteroids,” “colonoids” and “intestinal organoids.” Generally, “enteroids” are a type of organoid obtained from isolated intestinal crypts or stem cells of the intestine (16). Colonic counterparts are termed “colonoids.” Intestinal organoids should include enteroids and colonoids literally and some authors also use the term “intestinal organoids” and “enteroids” as synonyms without the subdivision of “colonoids.” In some research groups, “organoids” refer to the cultures that contain both epithelial and mesenchymal components, whereas the term “enteroids” has been suggested for 3D structures that contain only epithelial cells (17). Additionally, sometimes ‘intestinal organoids’ are specially referred to as 3D structures originating from inducible pluripotent stem cells (16). In this review, the term ‘organoids’ means any of these complex, multicellular 3D systems, the stem cell- or tumor cell-initiated clusters or aggregates. There are several other terms exclusively used in organoid research, some of which are also used in this review. “Assembloids” are organoids generated from the spatial fusion and functional integration of multiple cell types (18). “Tumoroids” means “tumor-like organoids” (19). “Mammospheres” refer to mammary epithelial stem cell aggregates but are also acceptable to indicate the aggregates derived from breast cancer cell niches or breast cancer cell lines (20). Therefore, we suggest that definitions for ambiguous terms used in organoid works should be defined in context to avoid misunderstanding.

Once the 3D structures assemble *in vitro* with highly ordered architecture, they can work as powerful models for disease investigations. The widespread applications of murine and human organoid models have accelerated the development of various areas in human medicine, such as developmental and stem cell biology, disease modeling and mechanism exploration, toxicology and drug screening, and personalized medicine (21–23) (Figure 1). In recent years, an increased number of organoid models were specifically designed to be investigated for veterinary medicine. Following the establishment of organoids, veterinary researchers benefited greatly from existing and emerging applications of organoid systems because organoid systems had greater experimental value and their 3D features contributed to a more in-depth understanding of organogenesis and morphogenesis than conventional 2D cultures. Compared with *in vivo* models, it is cheaper and easier to maintain and manipulate, and a more ethical alternative (24). In contrast to the predominant emphasis on disease models of cancer and chronic illnesses in human medicine, veterinary research places greater emphasis on infectious diseases and nutritional disorders. Thus, organoids demonstrate their unique efficacy because they can be tailored from specific species and tissues to investigate diseases of veterinary importance, given the extensive diversity of animal species. Certain critical information is necessary before starting any organoid culture. Essential considerations are to choose the appropriate organ or cells (tissue pieces, iPSCs, or ESCs) and the suitable culture protocol, extracellular matrix, growth factors, and morphogens [signaling molecules that act in the patterning of cells during embryonic development (25)]. Acquisition of in-depth knowledge pertaining to these considerations, together with an advancement in the protocols for culturing organoids, should create greater applicability of these 3D models in diverse fields. But in this review, owing to space constraints, the context mainly emphasizes applications for veterinary diseases and provides a framework for the emerging applications of organoid models particularly for veterinary diseases (Table 1). In this review, we highlight the currently known achievements and projects of disease modeling carried out in the pursuit of the welfare of livestock and companion animals.

**Table 1:** Use of organoid technology to investigate veterinary diseases.

Disease type	No.	Modeled disease	Species	Organ origin	Ref.
Infectious diseases	1	Caprine arthritis encephalitis	Goat	Mammary gland	(26)
	2	Porcine respiratory coronavirus infection	Pig	Airway	(27)
	3	Porcine epidemic diarrhea (PEDV)	Pig	Intestine (duodenum, jejunum, ileum and colon)	(28)
	4	Porcine deltacoronavirus infection	Pig	Intestine (anterior duodenum, jejunum, and ileum)	(29)
	5	Transmissible gastroenteritis (TGEV)	Pig	Intestine (jejunum)	(30)
	6	Feline infectious peritonitis (FIP)	Cat	Intestine (ileum and colon)	(31)
	7	Bovine Rotavirus (Group A Rotaviruses) infection	Ox	Intestine (ileum)	(32)
	8	Proliferative enteropathy	Pig	Intestine (ileum)	(33)
	9	Toxoplasmosis	Pig, Ox	Intestine (proximal jejunum)	(34)
	10	Salmonellosis	Pig, Ox	Intestine (proximal jejunum)	(34)
	11	Enterotoxigenic *E. coli* infection	Pig	Intestine (duodenum, jejunum without PP, ileum)	(35)
	12	Rabbit haemorrhagic disease	Rabbit	Intestine (duodenum, jejunum, and ileum), hepatobiliary tissue	(36, 37)
Inflammatory disease	13	Inflammatory bowel disease	Dog	Intestine (small intestine and colon)	(38, 39)
Cancer	14	Prostate cancer	Dog	Cancer	(40)
	15	Bladder cancer	Dog	Cancer	(41–43)
	16	Bladder cancer, mammary and skin tumors, lung cancer, and melanoma	Dog, Cat	Cancer	(44)
	17	Follicular cell thyroid carcinoma	Dog	Cancer	(45)
	18	Medullary thyroid carcinoma	Dog	Cancer	(46)
	19	Lung adenocarcinoma	Dog	Cancer	(47)
	20	Mammary tumor	Dog	Mammary tumor and non-neoplastic mammary tissue	(48)
Metabolic Diseases	21	Copper storage disease	Dog	Liver	(49, 50)
	22	Hepatic steatosis	Cat	Liver	(51, 52)

No., number; Ref., reference(s); PP, Payer’s Patch.

## 2. Organoid models for veterinary infectious diseases

Veterinary infectious disease is an intricate world where the pathogens of domestic animals may lead to suffering or death, massive economical losses, overuse or abuse of antibiotics in disease control, or cause zoonotic diseases in humans (53). Infectious disease is a result of a two-way interaction that happens between the hosts and microorganisms. The invasion of microbes is accomplished by a series of chronological steps, typically including access to a portal of entry into their hosts, recognition of target cells in the barrier system, colonization or breaching the barrier system, colonization of new populations of target cells (such as leukocytes and endothelial cells), invasion, systemic spread or invasion of a specific organ system, and eventually leading to systemic dysfunction manifest as disease (54). The capability of microbes to cause disease (their pathogenicity) is dominated by their ‘virulence factors’ carried by their genes, which are the consequences of the evolutionary adaptations to resistance factors expressed by hosts and creates a spectacular diversity of potential interactions. Typically, infectious disease outbreaks in herds occur only when several elements are present together. According to the model of ‘chain of infection’, every infection originates from the interaction between the host, pathogen, and environment, relies on the reservoir, portal of entry and exit, means of transmission, and ends with the infection of a new host (55). The complexity of each infectious disease poses a plethora of pathogen- and species-specific challenges to understand the pathogenesis and to develop prevention and control strategies.

In the past, *in vivo* animal models or *in vitro* monolayer cell culture were widely applied for the studies of pathogen biology and drug development (24). Unsurprisingly, both methods have obvious limitations. *In vivo* animal models have the structural diversity that maintains the physiological activities of the animals, but they are expensive and may pose ethical dilemmas. Conversely, *in vitro* monolayer cell culture is simple to manipulate, but lacks the spatial structure or microenvironment of tissue. Compared with conventional 2D cell culture and animal models, organoid technology is a more flexible and durable tool for modeling infections to study interactions between microbes and hosts, which is explained by features of providing better cellular differentiation and diversity with the presence of intercellular microenvironments. For example, enteroids from different intestinal segments still retain specific characteristics and show different degrees of adaptation to infections (28), even though all the cells are exposed to the same extracellular environment. Advancements in organoid technique have boosted progress in understanding the infectious disease or host-microbiome interactions and presents new opportunities for the discovery or development novel drugs for preventing and controlling infectious diseases.

To date and to the best of our knowledge, organoid technology has modeled 12 veterinary infectious diseases, including infections caused by viruses [caprine arthritis encephalitis virus (26), swine pulmonary and enteric coronavirus (27–30), feline coronavirus (56) and rabbit calicivirus (36)], bovine rotavirus (Group A rotaviruses) (32), bacteria (*Lawsonia intracellularis* (33), *Salmonella typhimurium* (34) and Enterotoxigenic *E. coli* (35)), and a parasite [*Toxoplasma gondii* (34)]. Detailed information on organoid models for specific infectious diseases is listed in Table 1.

The first organoid model for infectious disease is reported as early as 2005 (26). Goat mammary organoids were established from single mammary gland cells and treated with viral suspension into cultural supernatant as an approach to model caprine arthritis encephalitis virus (CAEV) infection. CAEV is a lentivirus of the family *Retroviridae* and causes arthritis, encephalitis, and/or pneumonia in adult goats (57). The 3D model of the goat mammary gland was a lobulated mammosphere-like or acinus-like structure consisting of luminal epithelial cells and peripheral myoepithelial cells limited by an outer layer of basal membrane, with responses to hormones and growth factors, which thus mimicked the structure and function of the mammary gland. The author complicated the model by importing peripheral blood leucocytes because the monocyte–macrophage system plays a critical role in the systemic spread of CAEV. However, the location and amount of the antigen were not specifically identified since there might be different levels of infection between epithelium and leukocytes. It is a clever design for veterinary infectious disease studies by enriching the complexity of livestock organoids through co-culturing epithelial organoids and immune cells (26).

The coronavirus disease 2019 (COVID-19) pandemic has exhibited widespread and disastrous social impacts on humankind. Similarly, coronaviruses in veterinary medicine also cause severe economic loss in farm animals and morbidity in companion animals. Porcine respiratory coronavirus (PRCoV), a naturally occurring spike deletion mutant of highly enteropathogenic transmissible gastroenteritis virus (TGEV), could be a surrogate to study the pathogenesis of human respiratory coronaviruses. Long-term 3D porcine airway organoids (AOs) derived from basal epithelial cells and AO-derived monolayer cultures were generated and provided insights into the pathogenesis and innate immunity of PRCoV (27). In these models, 3D AOs consists of four major types of airway epithelial cells identified by immunostaining, including ciliated cells, goblet cells, basal cells, and club cells. Especially, the ciliated cells, goblet cells, and club cells were mainly distributed on the surface of “tracheae spheres.” Ciliated cells also had two orientations either toward or reversed from the lumen. AO-derived monolayer cultures from 3D AOs offset limitations of 3D AOs to access the apical surface for pathogens. AO-derived monolayer cultures also expressed markers of ciliated cells, goblet cells, and basal cells even with more differentiation. Both AOs systems recapitulated the *in vivo* airway complex epithelial cellularity. 3D AOs and AO-derived monolayer cultures are susceptible to both PRCoV and TGEV despite the variable extent of permissiveness and can produce a pronounced IFN and inflammatory response to viral infection, which reflected well the events associated with PRCoV infection *in vivo*.

Recent publications regarding organoid applications in veterinary infectious diseases have shown a focus on gastrointestinal disease since 2019. Since the first generation of intestinal organoids of mice in 2009, intestinal organoids from intestinal crypts of humans and almost all categories of farm and companion animals (including the pig, ox, sheep, horse, chicken, dog, cat, and rabbit) have been developed by growing research groups (10, 58). The cellular diversity of intestinal surfaces offers the primary targets of many enteropathogenic pathogens, and the intestine can be a targeted site of attachment, invasion, and replication of many veterinary pathogens that mediate significant enteric pathological changes and clinical signs (59). Intestinal organoids containing these differentiated lineages of cell types including enterocytes, goblet cells, Paneth cells, enteroendocrine cells, and/or stem/progenitor cells, and recapitulating the extreme cellular heterogeneity and intercellular microenvironments, enables an improved understanding of several aspects of infectious processes such as adhesion, colonization, toxigenesis, invasiveness and local defense mechanisms.

In addition to species specificity due to the different pathogen’s tropism, the samples to generate the organoid model were mostly also obtained from animals of the most susceptible ages and from the most susceptible intestinal segments to capture classic features of the disease. For example, the infectious models of Porcine epidemic diarrhea (PED) or bovine rotavirus disease are mostly reported in newborn animals, so the scientists used 2- to 10-day-old specific pathogen free (SPF) piglets and colostrum-deprived neonatal calves, respectively. Ileum was used to generate organoids in the modeling of *Lawsonia intracellularis* infection because *L. intracellularis* are mainly distributed in that anatomic location (33). All enteroids are derived from intestinal crypts which include a pool of adult multipotent stem cells called crypt base columnar cells. One of the interesting distinctions is the initial step for the crypt treatments. Most study methods would isolate the tissue of the crypt or fractions with the highest ratio of crypts to villi to start three-dimensional enteroid cultures rather than single stem cell suspension. Tissue derived enteroids could also contain intermingled Paneth cells and supporting structures such as an extracellular matrix. One limitation in most of these studies is that the number of animals used to derive organoids was not stated and hence individual donor’s variation in developing the model is not accounted for. Additionally, in the modeling of *L. intracellularis* infection, only one segment of ileum from one 90 kg pig was used for organoid culture. Single animal derivations show the advantage of organoids in reducing animal use, but citing the sample size, or derivation of tissues from multiple animals to know more about how individual variations affect organoid generations and the repeatability of each protocol is relevant.

The intestinal epithelial cell is highly organized with the polarity of distinct apical and basolateral plasma membrane domains, which is critical for barrier formation and nutrient transport (60). Generally, intestinal organoids are buried in an extracellular matrix-containing scaffold which usually results in a polarized epithelium with an apical side facing the inside of the organoids (basal-out model). Unlike some pathogens, most enteric pathogens adhere and invade through the apical side of mucosal cells, although there are exceptions, for example *Listeria monocytogenes*, which exclusively invades the host *via* receptors on the basal side of intestinal epithelial cells even when the bacteria are on the surface of the intestinal mucosa (61). The basal-out organoid system limits the access that initiates the recognition and attack by pathogens. For example, it is recorded that basal-out organoids were not susceptible to TGEV infection (30). To solve this problem, there are several approaches. Firstly, pathogens can be imported to the lumen of the basal-out enteroid model by microinjection (55). However, microinjection poses technical and equipment thresholds, is also laborious and has the potential to damage the enteroid structure. Probably that’s why this method was seldom adopted in modeling veterinary infectious diseases. To expose the apical surface, organoids can be disrupted (enzymatically or mechanically) into smaller pieces and co-incubated with pathogens as with the organoid modeling of feline coronavirus, bovine rotavirus, *Toxoplasma gondii* and *Salmonella typhimurium* infection (32, 34, 56). This approach does cause the loss of polarity of the epithelium and devalue *in vitro* organoid models. Even so, disrupted enteroids will subsequently re-assemble into 3D basal-out structures again. Another popular approach is to dissociate organoids and generate a organoid-derived monolayer culture system with the apical side upwards (62). This approach was adopted in the organoid models of porcine coronaviruses, rabbit calicivirus, and *L. intracellularis* infections (28–30, 33, 36). Among them, enteroids were plated on transwell membranes after being mechanically disrupted and exposed apically to *L. intracellularis* (33). In this situation, the cells form a barrier that mimics the mucosal surface of the intestines. The polarity of intestinal organoids can also be inverted in suspension culture so that the apical membrane is kept outwards. This model permits easier access to the apical surface for pathogens and increases the available exposed surface without impeding proliferative states of the enteroids. A pioneering model of an apical-out organoid model which better resembles normal physiological features, characterized by an apical membrane on the surface, for TGEV infection was successfully developed. This approach to infection facilitates the study of swine enteric virus infection and the organoid is considered a next-generation porcine enteroid (30).

Organoid models can elucidate the cellular response to infection, previously limited by the absence of robust *in vitro* intestinal models. Porcine enteropathogenic CoVs (28–30), feline infectious peritonitis virus (FIPV) (31), rabbit hemorrhagic disease virus (RHDV) (36, 37), and *L. intracellularis* (33) all have similar issues of poor viral propagation or bacterial infections in conventional 2D models. Before organoids, available species-specific transformed and non-transformed epithelial cells had obvious drawbacks, such as poor viral propagation, lack of cellular heterogeneity, or the presence of genomic abnormalities (28, 63). Intestinal explants or primary cell cultures of intestinal epithelial cells recapitulate critical features of the *in vivo* organ, but they are not suitable for long-term culture due to limited viability (24–48 h) (63, 64). Fortunately, infectious models based on the species-specific organoids have improved these situations. Porcine enteroids been generated for porcine enteropathogenic CoVs including porcine epidemic diarrhea virus (PEDV) (28), porcine deltacoronavirus (PDCoV) (29) and transmissible gastroenteritis virus (TGEV) (30), and proved better *in vitro* models for further in-depth studies. Feline infectious peritonitis (FIP) is caused by mutated FeCoV, feline infectious peritonitis virus (FIPV) (31), with two serotypes of FCoV (based on antigenicity), types I and II. While both types may cause feline infectious peritonitis (FIP), type I is more common in domestic feline populations. The serotypes differ primarily in growth characteristics in cell culture and receptor usage, and most of the experimental work so far has worked on the Type II strains because Type I strains are difficult to grow well in cell culture. In 2020, a communication was published that was based on the feline intestinal organoid model, GFP-expressing recombinant serotype I FECV, which does not grow in available monolayer cell culture, can adapt to feline colon organoids, and persistently propagated. Although this pathogen cannot adapt to the enteroids from feline ileum, this result represents a new path *in vitro* that has the potential to study the mechanism of serotype I FECV infection, FIPV transition, and disease development *in vitro* (56). Rabbit hemorrhagic disease virus (RHDV) is a hepatotropic calicivirus and it has been hypothesized that it evolved from a benign, enterotropic calicivirus (65). Rabbit calicivirus *Australia-1*, an enterotropic lagovirus that does not grow in conventional cell culture, but can adapt to leporid hepatobiliary organoid-derived monolayer culture (37). However, this lagovirus also does not grow in rabbit intestinal organoid-derived cell monolayers (36). The reasons are putatively due to the lack of suitable conditions or any cofactors that are required for host-calicivirus crosstalk. For example, bile plays an important role in generating the human enteroid model of caliciviruses (noroviruses) (66), and it might be also critical for the rabbit counterpart.

In other studies, enteroids are used as novel *in vitro* models to identify the similar intestinal epithelial response with *in vivo* processes after infection, including infectious tropisms of specific intestinal segments and cell types, cytokine, and inflammatory responses in the gene transcriptions. *Toxoplasma gondii* is one of the most ubiquitous parasites and can infect almost all homoeothermic species including humans and livestock, being present in all countries (67). Infectious foci of *T. gondii* were detected in the generated porcine and bovine ileal organoids (34). In the same article, porcine and bovine ileal organoids were infected with *Salmonella typhimurium*, which is an important foodborne pathogen (68). The infection of *S. typhimurium* 4/74 in bovine enteroids resulted in the presence of luminal bacteria while bacteria was present at the periphery of porcine enteroids. Enterotoxigenic *E. coli* (ETEC) is one of the important causes of postweaning diarrhea in piglets which results in increased mortality, reduced growth rates, and severe economic losses in swine husbandry (35). Both porcine jejunal and ileal enteroid monolayers supported the F4-mediated adhesion of ETEC bacteria (35). They are all successful pilot studies for generating enteroid models for veterinary protozoal and bacterial research.

In a study of group A rotaviruses, important zoonotic pathogens that causes severe diarrhea in children and young animals including cattle (32), the authors revealed two ligands were required for rotavirus to enter small intestine epithelial cells in cattle. However, viral ingress into cells was also detected based on human enteroids without the two identified ligands, albeit less efficiently than bovine enteroids. In this study, enteroid models exposed the potentially undetected ligand-receptor molecular crosstalk that was not identified from other experimental methods and assays. The study demonstrates the critical role organoids can play in basic research.

So far, no fungal disease has been included in the list of published research articles using organoids for animal diseases. Applications of generating human epidermal organoids provides an *in vitro* model of dermatophyte infections, which has potentials to study pathogenesis of fungal infections and to test efficiency of antifungal drugs (69). Dermatophyte infections in humans and animals are similar, thereby facilitating the application of the model’s design principles to investigate fungal infections in an animal population. Failures in generating proper organoid models for studying veterinary diseases did and do occur. Even so, all the results demonstrated that the establishment of this new 3D cultivation system will facilitate applications in many different realms of veterinary infectious diseases. Successful developments of organoid fabrication techniques that can be more flexible and reliable to adapt to various veterinary infections and overcome current challenges are anticipated as knowledge continues to accumulate.

## 3. Organoid model for chronic inflammatory disease

Inflammatory bowel disease (IBD) identifies a spontaneously occurring group of chronic idiopathic enteropathies leading to serious debilitating inflammation of the gastrointestinal tract (70). Like IBD in humans, canine IBD (cIBD) is a multifactorial disease resulting from a combination of genetic predispositions, alterations of intestinal microbiota, and immunological aberrations in intestinal mucosa (70).

Among the large animal models used in studying multiple chronic human disorders including IBD, the canine model is particularly relevant based upon environmental and genetic similarities, analogs of gut anatomy, physiology and pathology, and resemblance of composition of gut microbiota (71–73). Canine intestinal organoids are a well-developed and characterized model for veterinary and translational research (38, 39, 70, 74–77). In addition to its capacity for epithelial differentiation, the canine intestinal organoid-derived monolayer offers an accessible tissue interface, exhibiting characteristics such as polarization, lineage-dependent differentiation, the formation of tight junction barriers, permeability, and the expression of crucial efflux pumps (74). Utilizing intestinal tissues/biopsies bearing cIBD to generate organoids offers an opportunity to study pathogenesis, features and possible therapeutic without sacrifice of living animals. Two studies generated canine intestinal organoids from dogs with IBD (38, 39). However, the histology and transmission electron microscopy revealed similarity between intestinal organoids derived from IBD and healthy dogs (38). Also, the expression of the EP4 prostaglandin-receptor (EP4R), a receptor that is involved in the pathogenesis of IBD and works a target of treatment, showed no significant differences by using the RNA *in situ* hybridization (ISH) probe (38). That might be due to the single epithelial lineage of constructed intestinal organoids. It is believed that organoids recapitulated genetic features of the tissue well, even after passages, which facilitates the study of transcriptomic profiles of intestinal organoids from dogs with inflammatory bowel disease after lipopolysaccharide (LPS) stimulation (39). LPS treatment revealed decreased expression of several cancer-associated genes and opposite expression patterns of anion transport, transcription and translation, apoptotic processes, and regulation of adaptive immune responses between IBD enteroids and colonoids. The organoid cIBD model provides new data describing the profiles of gene expression. Along with a diversified co-culture system involving other cell lineages (e.g., immune cells) and intestinal microbiota, the organoid model for cIBD could be well-developed and have a significant impact on screening drug options and discovering effective therapeutic methods for the disease with multifactorial nature both for humans and animals.

## 4. Organoids in veterinary cancer modeling

In cancer research, organoids have represented promising, near-physiological models of human cancers. Up to now, numerous robust and efficient establishment of human organoids can be achieved from various types of neoplasms arising from lung, breast, stomach, liver, pancreas, kidneys, urinary bladder, and prostate (19). In contrast, a limited but increased number of investigations of cancer organoids are emerging in veterinary oncology.

Organoid models of animal cancer, which might be derived from a small number of stem cells, enabled a better understanding of the molecular characteristics and neoplastic behavior of the disease in animals and acted as preliminary models for drug screening (78–81). For example, canine medullary thyroid carcinoma, studies on its biological behavior are scarce but treatment outcomes are always disappointing, so the development of a canine organoid model should help elucidate the best therapeutic approaches (46). Organoids could be formed from limited numbers of cells such as urine-derived stem cells, which are successfully used in the study of organoids of canine prostatic and urinary tract cancer (40–43). Availability of primary samples could hardly be an issue after adopting organoid culture systems for cancer research.

Due to better preservation of tumor microenvironment than conventional 2D cell line models, organoids of canine prostate cancer (40), canine bladder cancer (41–43), canine follicular cell and medullary thyroid carcinoma (45, 46), canine lung adenocarcinoma (48), and canine mammary tumor (47), accurately retain original morphological characteristics, genomic structures, and/or mutational profiles and recapitulate the genetic and phenotypic heterogeneity of the tumor cells. Organoids of canine prostate cancer (40) expressed epithelial markers (E-cadherin, CK5 and CK8), a myofibroblast marker (a-SMA) and a cancer stem cell marker (CD44). Established canine bladder cancer organoids (41–43) had a spheroidal structure and similar histology to naturally occurring bladder cancer in dogs. They were characterized by the expression of urothelial cell markers and resembled the cellular architecture of an invasive type of canine bladder cancer. Organoids derived from canine follicular cell thyroid carcinoma highly expressed thyrotropin receptor, sodium iodide symporter, pendrin, and thyroid peroxidase, the expressions of which are similar or higher compared to the primary tumors (45). Organoids derived from canine medullary thyroid carcinoma (46) showed similar histological features with the primary tumor after a long-term culture. Canine lung cancer organoids recapitulated the tissue architecture of canine lung adenocarcinoma, and expressed TTF1, a lung adenocarcinoma marker (48). Canine mammary tumor organoids recapitulated primary tissue structures and tumor characteristics such as cellular atypia, pleomorphism, and vacuolization, and sometimes squamous differentiation (47). Immunohistological features of the primary tissue from which they were derived were also retained including the molecular classification of their primary tumors with high fidelity in 82% of the cases (47). Moreover, tumors were also formed after the injection of the organoids into immune-deficient mice for canine prostate cancer, indicative of a similar neoplastic behavior (40). Genetic and genomic characteristics such as driver gene mutations, DNA copy number variations, and single-nucleotide variants were also conserved, even after extended passaging in canine mammary tumors (47).

Organoids are a proper model for *in vitro* drug assays with reproducible dose–response and expected dose-dependent tolerance. For example, after initial molecular characterization of established organoids, several genes could be found to be specifically upregulated, which could also be potential targets for novel therapies, as conducted in studying canine bladder cancer and canine lung cancer (41, 43, 48). Several genes including epidermal growth factor receptor (EGFR)/ERK signaling were upregulated in bladder cancer (41, 43). Trametinib, an inhibitor of ERK activation, showed extreme inhibition in cell viability of canine bladder cancer organoids along with the YAP inhibitor (43). Trametinib also decreased the xenografted growth of canine bladder cancer organoids in mice and enhanced sensitivity of the xenograft-derived organoids to carboplatin (43). Similarly, MEK pathway-related molecule expressions were also upregulated in canine lung cancer organoids (48). However, the sensitivity of canine lung cancer organoids or its xenografts to Trametinib was different among organoid strains, which indicated each organoid from different patient animals might show different individual-specific responses to treatment with a range of anticancer drugs, and which could be used to select individually-tailored treatment protocols. In the living biobank of canine mammary tumor organoids (47), PIK3CA-mutated organoid lines were more sensitive to an inhibitor of the PI3K/AKT pathway, alpelisib and tolerant to an inhibitor of the MDM2-TP53 interaction, nutlin-3a which has effects on other organoid lines, suggestive of a practical tool to investigate whether specific mutations predict therapy outcomes. In addition, canine mammary tumor organoids could also be genetically modified with a lentiviral vector or a customized canine CRISPR/Cas9 sublibrary and then were used to perform pooled CRISPR/Cas9 screening, where library representation was accurately maintained. The similarities in the drug responsiveness among the 3D *in vitro* models and the *in vivo* models (e.g., patient-derived xenografts) might largely be due to their similarities in enhanced cellular interactions via adhesion and secretion of soluble factors of tumors (30, 32).

Organoid-derived monolayer cultures, without losing their differentiated characteristics, was also performed to study veterinary tumors (42, 44). In the study of canine bladder cancer (42), organoid-derived monolayer cultures proliferated rapidly and had a similar sensitivity to anti-cancer drugs. Injecting monolayer organoid cells into immunodeficient mice also generated tumors with similar histopathological characteristics of urothelial carcinoma (42). More recently, direct monolayer cancer organoid models using animal tissues of dogs and cats were also generated by the same veterinary research group (44). The tissues including urine samples from bladder cancer diseased dogs, tissue samples from dog mammary tumors, melanoma, lung adenocarcinoma, cat skin tumor, and mammary tumors were directly used to generate monolayer organoid by special monolayer organoid media without formation of 3D organoid structure. The culture of direct monolayer organoids displayed constant passages and higher proliferation speed in the monolayer media. Direct monolayer organoids maintained the expression pattern of specific markers and demonstrated tumorigenesis *in vivo*. Furthermore, direct monolayer organoids showed concentration-dependent and different sensitivity to anti-cancer drugs among the different strains. These findings suggest that direct monolayer organoid culture methods can be used as a cheaper, easier, and less-time consuming research models instead of 3D organoids to study cancer biology and to expedite precision veterinary medicine.

Organoid cell culture approaches hold great potential and offer complex systems for various purposes in the field of cancer research, such as species-specific tumorigenesis and progression, and investigation of promising anticancer drug candidates and therapy combinations. In inoids equipped with the capacity to model tumor microenvironments. Cancer drugs can be tested by more complex tissue-like systems in the future, rather than by using conventional 2D cultures that do not fully manifest features of *in vivo* tumors. Organoid models can be an intermediate platform between conventional 2D cultures and the *in vivo* models. However, the generation of tumoroids is still more expensive and time-consuming to establish, maintain, and passage over conventional 2D cultures, which limits the current organoid model developments for veterinary cancer research (30, 32).

## 5. Organoid models for metabolic diseases

The available publications regarding organoid models for metabolic diseases are limited to canine and feline models. More precisely, we here discuss the relevance and limitations of veterinary 3D cultures to model and study metabolic dysfunctions associated with hepatic disorders in companion animals (canine congenital copper storage disease and feline hepatic steatosis) and discuss the feasibility of modeling the corresponding human diseases.

In dogs, mutations in the copper metabolism domain-containing 1 (COMMD1) gene lead to an autosomal recessive copper toxicosis associated with defective biliary excretion of copper resulting in massive hepatic copper accumulation and displaying many hallmarks of Wilson’s disease. The potential of hepatic organoid technology to address copper storage disease in the liver has been investigated in the COMMD1-deficient canine model recently (49). 14-day differentiated hepatic organoids grown from COMMD1^−/−^ dogs had a higher intracellular copper accumulation after being subjected to high copper levels for 3 h. This finding demonstrated liver organoids established from the dogs with an autosomal recessive COMMD1 deficiency maintain the defect of copper excretion, similar to the situation in the *in vivo* situation, and supported the feasibility of using diseased canine hepatic organoids to model the copper storage disease. Gene correction was performed on COMMD1-organoids by using lentiviral vectors bearing the COMMD1 gene. After transduction, COMMD1 gene supplementation normalized cellular copper content in the organoids to wild-type levels within 24 h evidenced by the copper level comparison after 3- or 24-h copper treatment with differentiated COMMD1^−/−^ and wild-type organoids. The authors indicated that these results confirm that organoids from canine liver diseases serve as a robust translational model for liver diseases such as Wilson’s disease and illustrated the amazing therapeutic potential for correcting genetic errors when combined with genome editing technology. In a follow-up study (50), the same group provided preclinical proof of concept for organoid-based cell transplantation *in vivo* with the hope that genetically corrected hepatic organoids could be a therapeutically relevant cell source for autologous transplantation for patients with metabolic liver diseases. They have documented the use of cells from autologous gene-corrected liver organoids for transplantation in the canine COMMD1-deficient models of copper storage disease. The results revealed that organoid-derived cells could be safely and repeatedly infused in a non-invasive manner *via* the portal vein with up to two-year survival post-transplantation as single cells although the translated organoid-derived cells were not fully mature and maintained functional integration *in vivo*. This preclinical study confirms the survival of genetically corrected autologous organoid-derived hepatocyte-like cells *in vivo* and warrants further optimization of organoid engraftment and functional recovery in a large animal model of human liver disease. Canine hepatic organoids provide platforms for pre-clinical modeling of liver diseases. The prospect of using hepatic organoids in cell therapy is encouraging but does require validation in the clinical setting. The development from liver stem cell cultures of the dog as an animal model is an important step to overcome the challenges of moving from basic translational research toward application in human patients.

Feline hepatic steatosis (FHS), one of the most common hepatobiliary diseases in cats, is characterized by triglycerides (TGs) accumulation in most of the hepatocytes, leading to significant hepatomegaly, impairment of liver function, and intrahepatic cholestasis. The pathophysiology of FHS is complex. Several similarities between feline and human steatosis and the unique sensitivity of this disease in cats encouraged scientists to establish a long-term feline model that might mimic Non-Alcoholic Fatty Liver Disease (NAFLD) and assess the efficacy of potential drugs for the treatment of FHS (51, 52). A spherical structure of the feline liver organoid model was generated with occasional epithelial folding and intraluminal projections and exhibited highly comparable transcriptomic or expressive signatures with hepatic adult stem cells or progenitor/biliary characteristics and differentiated potential toward hepatocyte-like cells. Under the circumstances of excess free fatty acids (FFA, including TGs), lipid accumulation was observed in organoids, and interestingly, organoids derived from feline liver accumulated significantly more lipid droplets than organoids derived from humans, indicative of a species difference. Moreover, differences in transcriptional activation between human and feline FFA-treated organoids were found which also reveals species differences in cellular lipid-metabolizing processes. In a follow-up study (52), the same authors used feline liver organoid models to test drugs for their potential to reduce lipid accumulation and they identified T863 and AICAR (diacylglycerol O-acyltransferase 1 inhibitor and adenosine monophosphate-activated protein kinase activator, respectively) as two promising candidates for further clinical evaluation used in the treatment of FHS. All these studies highlight the potential of organoids to model liver metabolic diseases and offer new perspectives in drug discovery to treat metabolic diseases (82).

## 6. Discussion

**Figure 2:**
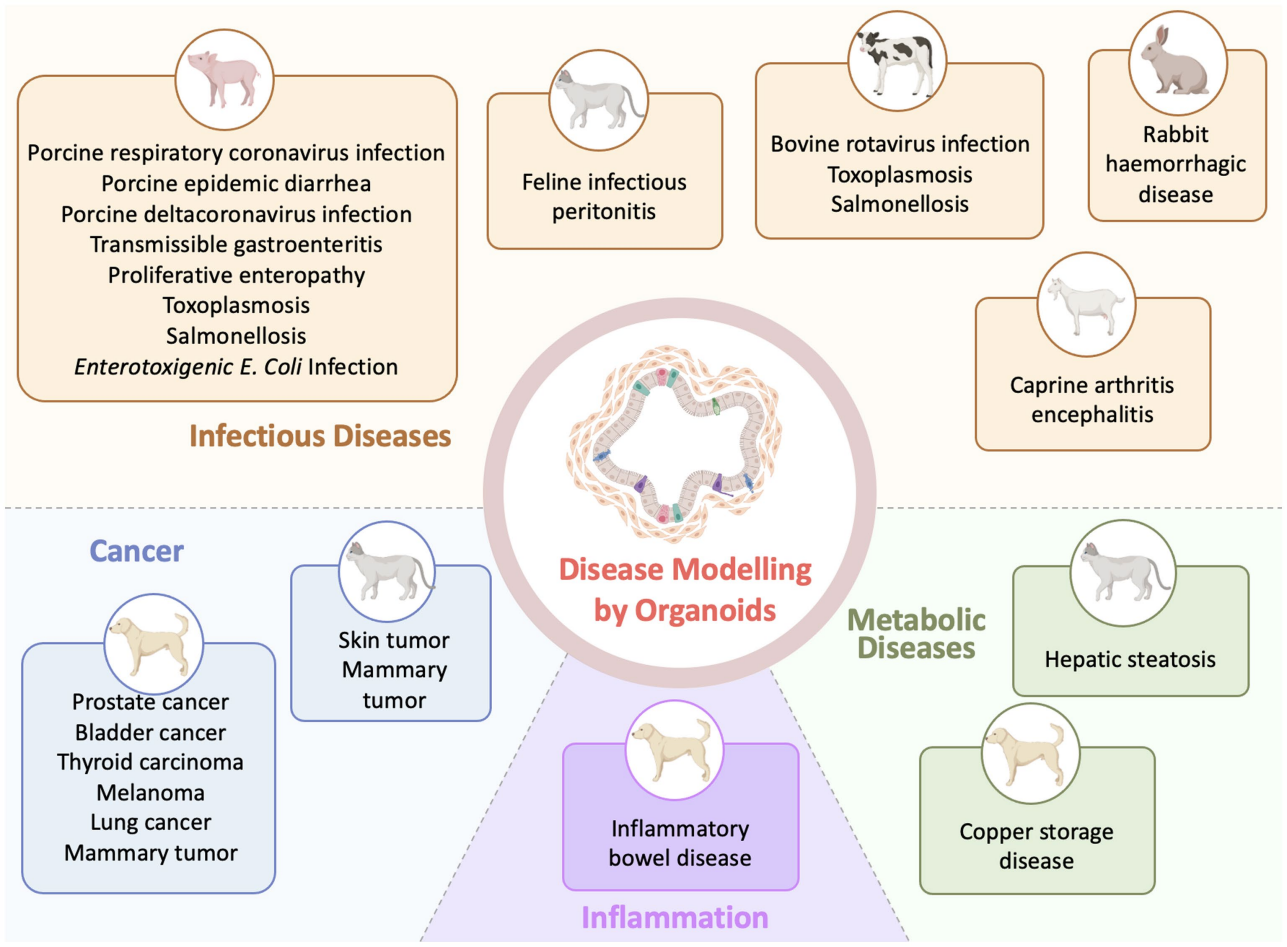
Applications of organoid technology in veterinary disease investigation (Created with BioRender.com).

Organoid models are being rapidly integrated into various aspects of veterinary research and are currently generated for diverse veterinary species and a diversity of organs due to improved derivation protocols and cultural conditions (58) (Figure 2). Such models, derived from primary tissues or immortalized cells, will pave the way for advanced *in vitro* applications in veterinary diseases. Compared with the number of generated veterinary organoid models from normal tissues, the applications of these organoids to model diseases are currently relatively scarce. For example, generating organoids for reproductive organs (ovaries, testes, oviducts, endometrium and placental) and embryoids made a lot of progress in farm animal species (83). But pathological models of organoids for reproductive organs are still few (83). The development of reproductive organoids under pathological conditions has the potential to offer novel therapeutic approaches and enhance interventions for addressing infertility in farm animals.

The future of the organoid disease model in the veterinary field is promising not only because of different existing veterinary organoids that can be manipulated but also due to the availability of powerful bioengineering methods like genome editing approaches. Because organoids are derived from stem cells, genome editing strategies provide ideal approaches to produce transgenic cells that possess key genotypes, which can then clonally expand and differentiate. Veterinary organoids with genome modifications can fundamentally contribute to modeling diseases with genetic anomalies (like cystic fibrosis), studying intricate receptor-ligand interactions for the infectious disease and malignant transformation (84). As talked above, gene correction was performed on COMMD1-cells with lentivirus, which normalized organoid capacity of copper storage, and attempted to achieve symptomatic remission for the transplanted patient dogs (49). Organoids are also amenable to gene strategies by gene knockdown by siRNA, shRNA, and CRISPR interference. As a result, organoid structures can be applications of genotype-to-phenotype research, congenital defect treatment and precision medicine purposes after genome manipulations.

Organoids assume a pivotal role within the domain of precision medicine. Some diseases, like cancer and IBD, are heterogeneous and include complex interactions of diverse cellular or non-cellular components. Organoids are cultivated utilizing pathological tissues procured from afflicted patients with features of micro-environments. Patient-derived organoids (PDOs) work as a tool for informed personalized medical determinations, with the capacity to anticipate patients’ reactions to therapeutic protocols and, in turn, foster the prospect of ameliorated treatment efficacy. In addition to personalized therapies, the establishment of a well-defined “living organoid biobank” for multifactorial diseases is gaining increasing attention. The critical advantage is to provide more accurate information of inherent intricacies of these diseases after large-scale sequencing and drug screening and more faithfully mirror patients’ receptivity to pharmaceutical agents and their capacity to endure drug-induced toxicities (85). As the concept of “One Medicine” suggests, therapeutic and technical methods can be shared between humans and animals for their mutual benefit but precision medicine in veterinary disease is still immature, and its application may vary depending on the species (86). Several veterinary organoid models also demonstrated the powerful potential of regenerative medicine (86). For example, corneal epithelial organoids in dogs and cats have successfully be cultured and maintained with expressions of cornea-specific epithelial and stem cell progenitor markers, which could be a new tool to model veterinary ophthalmology disease and test corneal drug and even further treat corneal diseases by corneal organoid transplantation or harnessing regenerative capabilities of limbal stem cells in the conception of regenerative medicine (87). The advantages of adopting a veterinary organoid system to model diseases and then applying it in precision and regenerative medicine are continually advancing, with potential benefits for both animal and human.

There is compelling evidence to suggest that pre-clinical studies or toxicity evaluation gain significant advantages from the adoption of organoid models (11, 88). Nonetheless, there is a limited amount of toxicity research conducted using veterinary organoid systems, like assessing the effect of chemicals in generated avian crypt-villus enteroids (89). The dog is the favored non-rodent mammalian animal model in pharmaceutical research, as endorsed by the FDA for gathering initial safety data on drugs intended for human use (90). A comprehensive protocol was provided for creating canine organoids in a dual-chamber system to form columnar epithelial monolayer with microvilli in the apical part of the cells in the permeable support enabling other researchers to determine the apparent permeability of therapeutic drug candidates (75). Preliminary findings pointed out promising potential in utilizing canine-derived organoid monolayers for conducting species-specific assessments of passive permeability concerning therapeutic drugs (77). Additionally, the utilization of porcine gastrointestinal organoid units has been proposed as a prospective *in vitro* tool with relevance to drug discovery and development (91). Animal gastrointestinal organoids can emerge to fill the gap between current other *in vitro* models and animal models to assess drug effectiveness and potential toxicity during preclinical investigations (38, 84). Moreover, once establishing disease modeling by using organoid systems have been achieved, integrating with ADME (adsorption, distribution, metabolism, and excretion) studies, can also be a crucial step in drug development, as integration will contribute to assessment of efficacy and safety of potential drug candidates in the context of the target human and veterinary diseases.

Recent organoid models for veterinary diseases were still imperfect and one of reasons was a lack of multicellular components of all lineages. All generated organoid models for veterinary diseases now are derived from adult stem cells (ASCs). ASC-derived organoids have greater differentiation features, but they solely consist of cells of a single lineage and do not have dynamic attributes and advanced functionalities of authentic organs, despite this arrangement offering the advantage of directly manifesting the impact of experimental treatments on specific target cells. However, the complexity of multicellular and dynamic organs always conceals more intricate mechanisms of diseases, and tumor microenvironments also play a crucial role in mediating some of the effects of chemotherapy and radiotherapy (92), so reinstating complexity and encompassing various components, including immune, neuronal, stromal, and vascular cells, along with physical and chemical microenvironments, as well as the microbiota, all within the context of the dynamic characteristics of a living system, presents considerable challenges in the field of veterinary disease modeling. This drawback could be partially overcome by establishing more complex co-culture organoid models or using iPSC/ESC-derived organoid culture systems, which also contain mesenchymal components. Other methods, like 3D-bioprinting and organ-on-a-chip models, might mimic more features of the whole living organs’ biological activities, dynamic mechanical properties, and biochemical functionalities as used in basic medicine, and their development is greatly encouraging. Organ-on-a-chip models or OrganoidChips are the innovative engineering approaches to create microfluidic cell culture devices for the production, precise control, and high-throughput analysis of organoids and their dynamic biomechanical microenvironment (84, 93). Furthermore, different organ-chip models can be fluidically linked to construct “body-on-a-chip” systems capable of simulating multiorgan interactions and functional responses at the systemic level (94). By adopting organ-on-a-chip organoid models, more advancements in veterinary fields, especially for disease modeling, will be achieved after better controlling microenvironment and mimicking tissue–tissue and multiorgan interactions (93). Once cellular heterogeneity in organoids is addressed in more sophisticated models, these models should help to better understand the pathophysiology of diseases and support the development of novel therapies, which also has the potential to greatly reduce the number of animal models used for equivalent purposes.

Admittedly, besides the reduction in complexity of current organoid models and its static nature, other significant limitations are also noticeable (95). First, organoids do not achieve the full maturity of *in vivo* organs. For example, the hepatic organoid exhibits the expression of markers associated with hepatic progenitor cells and biliary cells but without the mature hepatocyte marker HepPar-1 (51). Organoids usually model early developmental stages or specific cell type subsets, and achieving complete organ functionality remains a formidable challenge. As a result, the task of determining differentiation of organoids should be addressed in each research by assessing whether the cells within the organoids have developed into the desired specialized cell types or closely mimic the differentiation state of the organ of interest. To achieve this, the primary methods employed for examining organoid composition include assessing organoid morphology through techniques such as bright-field imaging, and light and electron microscopy. Additionally, immunofluorescence and immunohistochemical imaging, which can help provide insights into proportion of different cell types with the aid of specific cell marker antibody staining, is popularly used in veterinary medicine. For example, the cell types of intestinal organoids can be distinguished by specific markers for differentiation, including villin and villin1 for mature enterocytes, mucin 2 and mucin 5 ac for goblet cells, chromogranin A and synaptophysin for enteroendocrine cells, lysozyme for Paneth cells, Ki67 for proliferating cells, Lgr5, SOX9, and SMOC2 for intestinal stem cells, in addition to ZO-1 for apical proteins and β-catenin for basal proteins (28–30, 32–36, 56). Also, in veterinary organoid modeling research, it often begins with real-time PCR for quick and quantitative assessment of marker genes indicating cell identity, including transcription factors and differentiation markers. To delve deeper, western blotting can also offer insights into protein abundance, degradation, interactions, and post-translational modifications, revealing specific signaling pathway activities in committed cell types (96). For a more comprehensive view, high-throughput scRNA-seq analysis profiles all organoid cell types, both undifferentiated and committed, at the whole-genome transcriptome level (96). These profiles are then compared to cells freshly isolated from corresponding tissues or organs to assess the similarity of each cell population. This approach is particularly useful for understanding the diversity of cell differentiation states within cultured organoids, which often contain various immature cell types. Secondly, there are no standardized criteria of protocols for organoid establishment and quality control. Owing to diversity between individuals and protocols, outcomes vary from group to group. Organoid units even in the same culture system are heterogeneous in terms of viability, size, and shape, impeding phenotype screens. In some cases, the heterogeneity of organoids might mimic the real situation *in vivo* better than a highly stereotypic limited response. Standardization is an important step to ensure results obtained from organoid models are consistent, reproducible, and comparable. While there has been limited focus on standardization within veterinary domains, a publication has addressed this gap by establishing standardized protocols for maintaining 3D canine hepatic and intestinal organoid cultures (76). This effort aims to provide a reliable foundation for canine organoid culture procedures in biomedical research, promoting intradisciplinary sharing of knowledge. Third, organoids typically grow in a 3D structure with a substance called Matrigel as their microenvironment. The precise influence of Matrigel on the behavior of these organoids remains uncertain. It’s important to realize that Matrigel could impact how we use organoids for personalized medicine or organ-based cell transplantation. Future research should focus on understanding and addressing this issue. Lastly, organoids are relatively costly compared to traditional cell cultures.

Although organoid models are relatively expensive and cannot model the whole organism as an animal model does, they still cheaper and easier to manage than animal models and enable scientists to collect data quickly, while avoiding the ethical issues involved in using animals for research (24). Modeling different diseases by using organoid systems helps to replace animal experimentation in accordance with the 3R principle, i.e., the replacement, reduction, and refinement, and also 4R principle with responsibility of animal experimentation (97, 98). The concept of ‘One Health’ represents an advancement or evolution from the previous paradigm of ‘One Medicine’ with the incorporation of the ecosystem health (99). The versatility of organoid models in accommodating various species makes them notably significant within the ‘One Health’ framework to manage emerging and re-emerging zoonoses and elucidate the interconnected pathophysiological dynamics among human, animal, and environmental health (97). On the other hand, most cultures are genetically stable, can be propagated indefinitely, and can be frozen for storage in much the same manner as immortalized cells, thus providing ease of use, storage, and transfer. In a long-term study, enteroids could live normally for 4 months to more than 1 year with no or very few anomalies in their growth, morphology, and genetic profiles (29). In contrast, other, non-transformed *in vitro* models, such as primary cells or tissue explants, have a finite replication capacity and rapid senesce (100).

To conclude, organoid culture offers more than conventional 2D cell culture systems do in basic and applied research. The current application of organoids in veterinary diseases is in its infancy but developing quickly. Organoid models have a huge potential to be durable *in vitro* models for studying disease pathogenesis and drug development. The future of organoid technology in veterinary disease modeling is promising.

## Author contributions

BC wrote the first draft of the manuscript. RS and SG contributed to manuscript revision, edited, and approved the submitted version. All authors contributed to the article and approved the submitted version.
